# Erectile Dysfunction Drugs Changed the Protein Expressions and Activities of Drug-Metabolising Enzymes in the Liver of Male Rats

**DOI:** 10.1155/2016/4970906

**Published:** 2016-10-09

**Authors:** Salah A. Sheweita, Mona Wally, Mostafa Hassan

**Affiliations:** ^1^Biotechnology Department, Institute of Graduate Studies & Research, Alexandria University, 163 El Horreya Avenue 21526, P.O. Box 832, Alexandria, Egypt; ^2^Department of Environmental Studies, Institute of Graduate Studies & Research, Alexandria University, Alexandria, Egypt

## Abstract

Erectile dysfunction (ED) is a major health problem and is mainly associated with the persistent inability of men to maintain sufficient erection for satisfactory sexual performance. Millions of men are using sildenafil, vardenafil, and/or tadalafil for ED treatment. Cytochrome P450s (CYPs) play a central role in the metabolism of a wide range of xenobiotics as well as endogenous compounds. Susceptibility of individuals to the adverse effects of different drugs is mainly dependent on the expression of CYPs proteins. Therefore, changes in activities of phase I drug-metabolising enzymes [arylhydrocarbon hydroxylase (AHH), dimethylnitrosamine N-demethylase (DMN-dI), 7-ethoxycoumarin-O-deethylase (ECOD), and ethoxyresorufin-O-deethylase ((EROD)] and the protein expression of different CYPs isozymes (CYP1A2, CYP2E1, CYP2B1/2, CYP3A4, CYP2C23, and CYP2C6) were determined after treatment of male rats with either low or high doses of sildenafil (Viagra), tadalafil (Cialis), and/or vardenafil (Levitra) for 3 weeks. The present study showed that low doses of tadalafil and vardenafil increased DMN-dI activity by 32 and 23%, respectively. On the other hand, high doses of tadalafil, vardenafil, and sildenafil decreased such activity by 50, 56, and 52%, respectively. In addition, low doses of tadalafil and vardenafil induced the protein expression of CYP2E1. On the other hand, high doses of either tadalafil or sildenafil were more potent inhibitors to CYP2E1 expression than vardenafil. Moreover, low doses of both vardenafil and sildenafil markedly increased AHH activity by 162 and 247%, respectively, whereas high doses of tadalafil, vardenafil, and sildenafil inhibited such activity by 36, 49, and 57% and inhibited the EROD activity by 39, 49, and 33%, respectively. Low and high doses of tadalafil, vardenafil, and sildenafil inhibited the activity of NADPH-cytochrome c reductase as well as its protein expression. In addition, such drugs inhibited the expression of CYP B1/2 along with its corresponding enzyme marker ECOD activity. It is concluded that changes in the expression and activity of phase I drug-metabolising enzymes could change the normal metabolic pathways and might enhance the deleterious effects of exogenous as well as endogenous compounds.

## 1. Introduction

The male erectile dysfunction (ED) is a common and multifactorial disease, which strongly impairs the quality of man's life. According to the Massachusetts Male Aging Study, ED affects more than 150 million men worldwide and 50% of them are between the ages of 40 and 70 years [1]. Phosphodiesterase-5 inhibitors (PDE5Is) become the first-line therapy for millions of men suffering from ED. PDE5 inhibitors are structurally similar to those of the cGMP and competitively bind with PDE5 leading to inhibition of the cGMP hydrolysis. Therefore, accumulation of cGMP enhances the levels of nitric oxide (NO), which in turn activates guanylate cyclase to produce more cGMP, leading to smooth muscles relaxation of the corpus cavernous tissue and prolonged erection process [2]. The three EDDs (sildenafil (Viagra), tadalafil (Tadalafil), and vardenafil (Vardenafil) are well tolerated and effective, and the major clinical differences among them are the onset and duration of action providing treatment options for men with ED [3, 4].

The cytochrome P450s (CYPs) are superfamily of hemoproteins, which metabolise various compounds. The currently known CYPs in humans are classified into 18 different families and 44 subfamilies according to their amino acids sequence homology [5]. CYPs are widely accepted to be the key enzymes in cancer etiology and treatment, as they participate in the inactivation and activation of many anticancer drugs. They also mediate the metabolic activation of a vast amount of procarcinogens. The tobacco smoke contained N-nitrosamines that are metabolised to genotoxic products by different P450 enzymes particularly P450 2E1 and 2A6 [6]. Dimethylnitrosamine (DMN) is metabolised by DMN-N-demethylase I leading to the generation of carbonium ion that methylates DNA and other macromolecules. Furthermore, CYP2E1 isozyme is able to produce reactive oxygen species (ROS), leading to oxidative stress resulting in cytotoxicity [7]. Polycyclic aromatic hydrocarbons (PAHs) and heterocyclic aromatic amines (HAAs) are other potent carcinogens for animals and humans. These compounds are usually activated by cytochrome P450 enzymes (1A2, 1A1, and 1B1) [8]. The CYP1A-dependent aryl hydrocarbon hydroxylase (AHH) activity activates benzo(a)pyrene (B(a)P) into 7,8-diol-epoxides which covalently bind to DNA and initiate cancer [6, 9]. In addition, PAHs are exogenous ligands that directly bind to the aryl hydrocarbon receptor (AhR) and activate them leading to induction of gene expression of CYP1A1 and 1B1. This explains how AhR plays a key role in cigarette smoke-induced lung cancer [10, 11]. In addition, CYP1A2 takes part in the metabolism of some medications such as acetaminophen, imipramine, propranolol, clozapine, theophylline, and caffeine [12].

Sildenafil and vardenafil both are metabolised primarily in the liver via the cytochrome P450 (CYP) pathway, mostly by CYP 3A4 and to a lesser extent by CYP 2C9, while tadalafil is metabolised almost solely by the cytochrome P450 3A4 [13, 14]. The metabolism of these drugs was inhibited by concomitant administration of potent CYP3A4 inhibitors, such as azole antifungal and macrolide antibiotics. Coadministration of 3A4 inhibitors with the EDDs may elevate and prolong their serum concentrations and enhance their pharmacological and toxicological effects. Conversely, inducers of CYP 3A4 such as phenobarbital, phenytoin, and carbamazepine increase the clearance of EDDs and decrease their plasma concentrations. Therefore, the present study investigates the alternatives in the expression of various CYP isozymes (CYP1A2, 2B1/2, 3A4, 2C23, 2C6, and 2E1) and their associated enzyme activities including DMN-N-demethylase I, aryl hydrocarbon hydroxylase, ethoxyresorufin-O-deethylase, ethoxycoumarin-O-deethylase, and NADPH-cytochrome c reductase after treatment of rats with either low or high doses of tadalafil, vardenafil, and/or sildenafil. Inhibition or induction of the expression of various CYP isoforms and their corresponding enzyme activities could provide valuable information regarding the metabolic fate of a vast array of drugs and in the prediction of both drug-drug interactions and different responses to various drugs.

## 2. Materials and Methods

### 2.1. Chemicals

Vardenafil (vardenafil 20 mg), sildenafil (sildenafil 100 mg), and tadalafil (tadalafil 20 mg) are manufactured by Bayer®, Pfizer®, and Lilly® pharmaceutical Cos., respectively. Western blotting detection kit, primary antibody anti-CYP 2E1, 3A4, 2C23, 1A1, and 2B1/2, and anti-rabbit secondary antibody were purchased from ABCAM® pharmaceuticals Ponceau S stain; benzo(a)pyrene, ethoxycoumarin, cytochrome c, sodium dithionite, and all other chemicals were obtained from Sigma Chemical Co., St. Louis, MO, USA.

### 2.2. Animal Treatments

This study was carried out in strict accordance with the recommendations in the Guide for the Care and Use of Laboratory Animals of the College of Medicine, Alexandria University. The protocol was approved by the Committee of Postgraduate Studies & Research on the Ethics of Animal Experiments of the University of Alexandria. Surgery was performed under diethyl ether anesthesia, and all efforts were made to minimize suffering. Rats were housed in stainless steel wire bottom cages placed in a well-ventilated animal house, maintained for one week for acclimatization period on food and water ad libitum, and subjected to the natural photoperiod of 12 hours light : dark cycle. Rats were housed in standard cages and given food and water ad libitum. Rats were divided into eight groups (10 rats each). EDDs were suspended in distilled water and administered orally as repeated doses for 21 days (Table 1).

### 2.3. Enzymes Assay

At the designated time point, the thoracic cavity of rats was opened for the whole body. Liver tissues were vigorously washed in an iced solution of 0.25 M sucrose, which contained 0.001 M EDTA, to avoid contamination from erythrocyte-containing enzymes. Liver tissues were homogenized in 3 volumes of 0.1 M phosphate buffer, pH 7.4, and subsequently centrifuged at 10,000 ×g for 15 min at 4°C. The supernatant was subsequently centrifuged at 105,000 ×g for 60 minutes at 4°C to sediment microsomal pellets, which were finally suspended in 0.1 M phosphate buffer, pH 7.4, and kept at −80°C until used.

Total microsomal protein content was determined according to the method of Lowry et al., 1951 [15], using BSA as a standard protein. Total microsomal CYP and cytochrome b5 were measured according to the method of Omura and Sato, 1964 [16], using the molar extinction coefficients 91 and 185 mM^−1^ cm^−1^, respectively, for the reduced CYP-CO complex and reduced cytochrome b5, respectively. NADPH-cytochrome c activity was assayed according to the method of Williams Jr. and Kamin, 1962 [17], using extinction coefficient 21 mM^−1^ cm^−1^ for calculation of the activity.

Microsomal NDMA-dI activity was determined according to the method of Venkatesan et al., 1968 [18], with the modifications of Mostafa and Sheweita, 1992 [19]. Substrate concentration was 4 mM NDMA, which represents the saturation level for NDMA-dI. The amount of formaldehyde formed was determined. The enzymatic activity of NDMA-dI was expressed as nmole of formaldehyde per mg protein per hour. Microsomal aryl hydrocarbon hydroxylase (AHH) activity was determined according to the method of Wiebel and Gelboin, 1975 [20]. Briefly, the volume of the incubation mixture was 1 mL containing 50 mM Tris-HCl buffer, pH 7.4; 3 *μ*mole MgCl_2_; 0.6 *μ*mole NADPH; 100 nmole benzo(a)pyrene; 0.1 mL of microsomal protein (10 mg/mL). The reaction was incubated at 37°C for 10 min. The reaction was terminated by the addition of 1 mL acetone. The 3-hydroxy benzo(a)pyrene was extracted with 2 mL hexane. The fluorescence intensity was measured at excitation and emission wavelengths of 396 and 522 nm, respectively. Ethoxycoumarin hydroxylase activity was assayed by the method of Greenlee and Poland (1978) [21]. The intensity of 7-hydroxycoumarin fluorescence was measured at excitation and emission wavelengths of 338 and 458 nm, respectively. Ethoxyresorufin-O-deethylase activity was determined by the methods of Pohl and Fouts, 1980 [22]. Product concentration was interpolated from a standard curve for resorufin.

### 2.4. Western Immunoblotting

The protein concentration of the pooled samples was determined according to the method of Lowry et al., (1951) [15]. Twenty micrograms of microsomal proteins from each pooled group was mixed with the sample application buffer (SAB), then boiled for 3 minutes, and loaded on a 10% SDS-polyacrylamide gel. After electrophoresis, proteins were transferred to nitrocellulose membranes using a semidry trans-blotter. After completion of trans-blotting process, the membranes were washed three times with TBS buffer pH 7.3 (8 g NaCl, 0.2 g KCl, and 3 g Tris-base/1 liter) for 10 minutes. After those membranes were incubated with 5% fat-free dry milk TBS buffer for 1 h at room temperature and then washed in TBST buffer (phosphate buffered saline containing 0.1% Tween 20) for 5 min and then in TBS buffer twice for 10 min, then, membranes were incubated for 2 hours using primary antibodies for CYPE1, 3A4, 2C23, 2C6, 2B1/2, and 1A1 using the dilution of 1 : 1000 and then washed twice using Tween-TBS (0.2 mL Tween/liter TBS) for 20 min, then with TBS for 15 min. All primary antibodies are purified immunoglobulin IgG suspended in buffered aqueous solution. After those membranes were incubated with secondary antibody-HRP using dilution of 1 : 7000 in TBS, then they were washed twice with Tween-TBS for 15 min followed by washing with TBS twice for 15 min. An ECL kit was used and the proteins expression of different CYPs isozymes was detected using X-ray film. The band intensity was measured using quantity one software program.

### 2.5. Statistical Analyses

SPSS software package (Version 17.0) was used for statistical analysis. The differences between means were analyzed using one-way analysis of variance (ANOVA) and the differences between means of all groups were tested using Least Significant Difference (LSD). Probability value less than 0.05 was considered statistically significant.

## 3. Results

**Figure 1:**
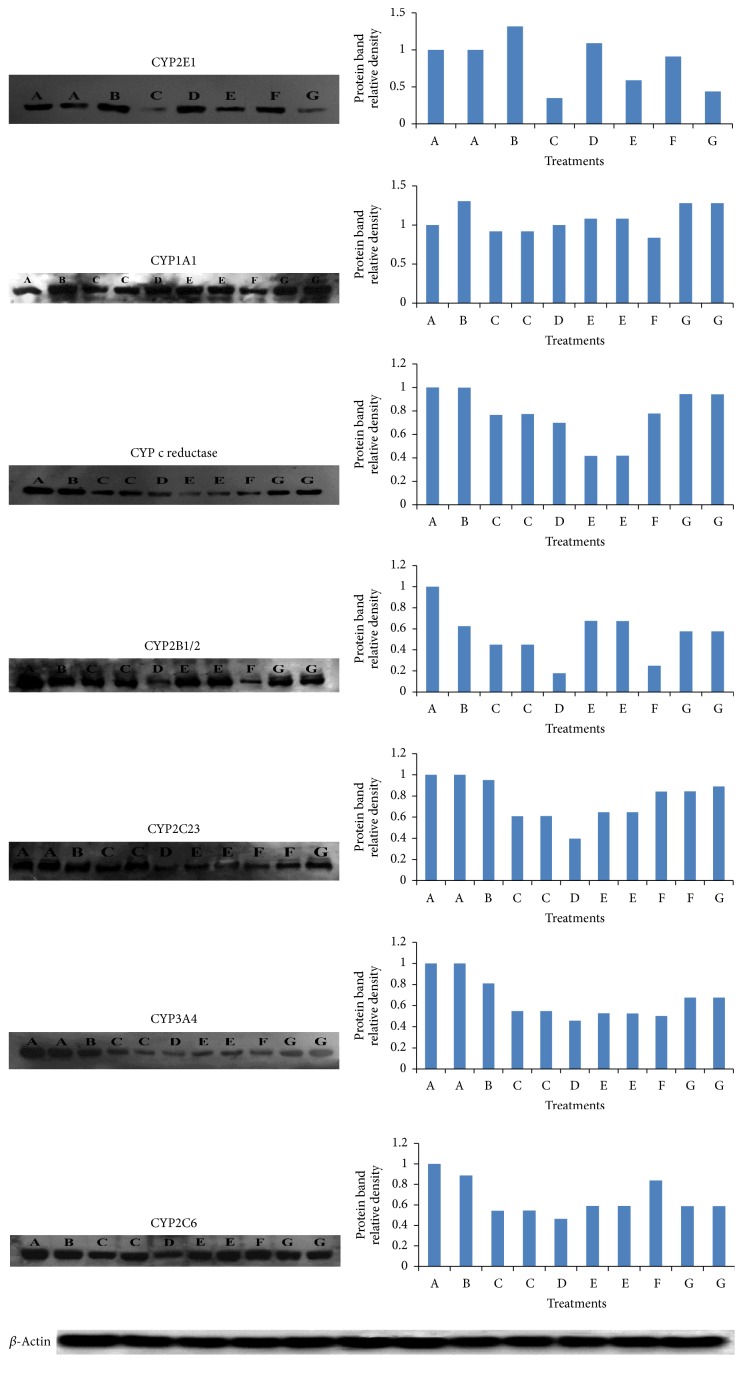
Western immunoblotting showed the influence of tadalafil, vardenafil, and sildenafil on the expression of CYP2E1, CYP1A2, cyt. c reductase, CYP2B1/2, CYP3A4, CYP2C23, and CYP2C6, respectively. Lane A is microsomal protein of matched control groups. Lanes B and C are microsomal proteins of rats treated with low and high doses of tadalafil; lanes D and E are microsomal proteins of rats treated with low and high doses of vardenafil; lanes F and G are microsomal proteins of rats treated with low and high doses of sildenafil, respectively.

The present study showed that low doses of tadalafil and vardenafil caused significant (*P* < 0.001) increase in the DMN-dI activity by 32 and 23%, respectively (Table 2). On the other hand, high doses of tadalafil, vardenafil, and sildenafil caused significant (*P* < 0.001) decrease in the DMN-dI activity by 50, 56, and 52%, respectively (Table 2). In addition, low doses of tadalafil and vardenafil induced the protein expression of CYP2E1 (Figure 1). However, such expression was inhibited after treatment of rats with the daily high dose of tadalafil, vardenafil, and sildenafil compared to control group (Figure 1). It was observed that high doses of either tadalafil or sildenafil are more potent inhibitors of CYP2E1 expression than vardenafil, which is consistent with the results of DMN-dI activity (Table 2 and Figure 1). Moreover, low doses of both vardenafil and sildenafil markedly increased the AHH activity by 162 and 247%, respectively (*P* < 0.001) (Table 2). On the other hand, high doses of tadalafil, vardenafil, and sildenafil significantly (*P* < 0.001) inhibited the AHH activity by 36, 49, and 57%, respectively (Table 2). In addition, the expression of the hepatic CYP1A2 was changed with different intensities under the influence of the EDDs (Figure 1).

Low and high doses of tadalafil, vardenafil, and sildenafil decreased the NADPH-cytochrome c reductase activity as well as its protein expression (Table 2 and Figure 1). In addition, such drugs inhibited the expression of CYP1B1/2 along with its corresponding enzyme marker, 7-ethoxycoumarin-O-deethylase (ECOD) activity (Table 2 and Figure 1). Low dose of tadalafil increased EROD activity by 24%, whereas sildenafil decreased such activity by 28% (Table 2). On the other hand, high doses of tadalafil, vardenafil, and sildenafil caused significant (*P* < 0.001) inhibition of the EROD activity by 39, 49, and 33%, respectively (Table 2).

Western immunoblotting of the most clinically important CYP isoforms revealed that low and high dose treatment of male rats with tadalafil, vardenafil, and sildenafil inhibited the expression of CYP3A4 and CYP2C23 with different intensities (Figure 1). In particular, vardenafil showed a potent inhibition of CYP3A4 expression compared to control group. These drugs also altered the expression of CYP2C6 (Figure 1).

## 4. Discussion

N-Nitrosamines are metabolised predominantly by DMN-N-demethylase I (DMN-dI) enzyme-CYP2E1 dependent in order to exert their cytotoxic and carcinogenic effects [19, 23]. It is well known that induction of DMN-dI activity could lead to the production of more reactive carbonium ions that alkylate DNA and other macromolecules [23, 24]. On the other hand, inhibition of CYP2E1 expression was effective in protecting the liver against the toxicity of a wide variety of toxic agents including carcinogens [25]. Ye et al., 2012 [26], reported that CYP2E1 induction increased oxidative stress, released high levels of inflammatory cytokines, and induced carcinogenesis. In accordance with this finding, inhibition of the CYP2E1 expression after treatment of rats with high doses of EDDs might significantly decrease the generation of reactive oxygen species (ROS) and could also protect the liver and probably other organs from cytotoxic and genotoxic effects of carcinogenic compounds [27]. Supporting this finding, it has been found that inhibition of CYP2E1 and DMN-dI activity played a significant role in decreasing the tumorigenicity and the carcinogenicity caused by N-nitrosamines [23, 28, 29]. Moreover, inhibitors of DMN-dI activity by diallyl sulfide (DAS) were found to decrease the level of DNA adducts (N7, O6-methyldeoxyguanosine) and consequently inhibited the tumorigenicity in different organs of mice pretreated with nitrosomethylbenzylamine, N-methyl-N-nitrosourea, and/or N-nitrosodimethylamine [30, 31].

Benzo(a)pyrene predominantly metabolised by the aryl hydrocarbon hydroxylase (AHH) into ultimately carcinogenic products [8, 30]. It has been reported earlier that the activity of AHH is mainly dependent on cytochrome P450 1A1/2 content [23, 30]. The present study showed a remarkable increase in the AHH activity after treatment of rats with low doses of either vardenafil or sildenafil, whereas high doses of such drugs inhibited the activity of this enzyme. In addition, low dose of tadalafil and vardenafil induced the expression of CYP1A1. The mechanism of induction of the expression of CYP1A1 might be due to induction of the aryl hydrocarbon receptor (AhR) [9, 31]. Moreover, induction of CYP1A2 is implicated in colon and lung cancers [32, 33]. On the other hand, inhibition of AHH may increase the bioavailability of the parent compound and may decrease its elimination. Moreover, low dose of sildenafil reduced the expression of CYP1A1 and may decrease the clearance of CYP1A1 substrates such as theophylline and caffeine, aromatic amines, and food-derived mutagens [12]. This inhibition might alter the metabolism of benzo(a)pyrene and other procarcinogens such as aryl arenes, nitroarenes, and arylamines that are dependent on the level of this isozyme. Interestingly, treatment of rats with low dose of sildenafil reduced the expression of CYP1A1, whereas high dose markedly induced such expression. With respect to other previous studies, the expression of CYP1A2 is not consistent with the AHH activity; this conflict may be due to the involvement of many other CYP isoforms such as 1A1, 1B1, and 3A4 in the metabolism of B(a)P [34, 35]. In accordance with the present study, it has been found that diallyl sulfide exerted an inhibitory effect in the colon and renal carcinogenesis in rats and also in human epidermal keratinocytes exposed to benzo(a)pyrene which might be due to inhibition of CYP1A1 [36, 37]. In addition, the genotoxicities of bone marrow and mammary tumors in mice caused by benzo(a)pyrene and 7,12-dimethylbenzo(a)anthracene, respectively, were reduced after treatment with diallyl thioesters, selenium-enriched garlic, and black tea [38–41].

It has been reported that EROD activity is the catalytic monitor of CYP1A1 since CYP1A1 preferentially metabolises ethoxyresorufin into resorufin [42, 43]. The present study showed that high doses of EDDs significantly inhibited the expression of CYP1A1 and its associated EROD activity. Inhibition of CYP1A1 expression could protect the liver and other organs against cancer progression. Supporting our suggestion, Rodriguez and Potter found that CYP1A1 induction promotes breast cancer proliferation and progression [44]. In parallel with this finding, epidemiological studies showed an inverse association between a higher intake of flavonoids and breast cancer risk which was attributed to selective inhibition of flavonoids to CYP1A1 expression [45, 46]. The expression of CYP2B1/2 and its associated ECOD activity represents the counterpart of human CYP2B6. The present data showed that low and high doses of EDDs inhibited the expression of CYP1B1/2 and its associated ECOD activity. This inhibition may increase the bioavailability of a wide range of CYP1B1/2-dependent drugs including antidepressants, anesthetics, and anticancer agents which may subject patients to drug-drug interactions [47]. The mechanism of inhibition might be due to the presence of methylenedioxyphenyl (MDP) group on EDDs which exerted high ability to bind and inhibit the protein expression of CYP3A4 [48] since low and high dose treatment of male rats with tadalafil, vardenafil, and sildenafil inhibited the expression of CYP3A4. In agreement with the present study, it has been found that sildenafil was a weak inhibitor of CYP3A4 [48, 49]. In addition, the previous study showed that tadalafil reversibly inhibits CYP3A4-, CYP2C9-, CYP2C19-, and CYP1A2-mediated metabolism either competitively or noncompetitively [50].

Sildenafil is metabolically activated by CYP3A and is converted to an active metabolite, N-desmethyl sildenafil, that has similar properties to the parent drug [51]. Korzekwa et al. (1998) evaluated two binding sites in CYP3A which can bind to two substrate molecules simultaneously [52]. The two binding sites have been used to explain the activation and substrate inhibition and biphasic saturation curves for the cytochrome P450 enzymes [52]. The mechanism of substrate inhibition was mainly due to the presence of the two sites either neighboring or at a distance away from the active site. It has been shown that one site is favorable for oxidation while the other site is less favorable or nonproductive [53]. When the substrate binds to the inhibitory site, the inhibited ternary complex (SES) is less capable of converting substrate to a product than binary complex (ES) [53]. Thus, if both binding sites of CYP3A are occupied with substrates at high concentration, the velocity of enzymatic reactions was underestimated by a loss in its activity [54]. This mechanism may explain the inhibitory effects of sildenafil, vardenafil, and tadalafil at high doses to different CYPs expression.

CYP2C6 of rats and its human counterpart CYP2C9 have a significant pharmacological importance in drug metabolism [55]. In the present study, low and high dose treatments of rats with either vardenafil or tadalafil moderately altered the expression of CYP2C6. In addition, low and high dose treatment of rats with vardenafil and/or sildenafil caused significant inhibition of CYP2C23 expression. It is well known that CYP2C23 catalyzes epoxidation of arachidonic acid to 11,12-epoxyeicosatrienoic acid (EET) which has an important physiological function [56]. Therefore, inhibition of CYP2C23 expression could reduce the production of EET and decrease omega-3 fatty acids metabolism leading to loss of their vasodilatory, anti-inflammatory, and cardioprotective effects [57]. The mechanism of inhibition of CYP 2C23 and probably other CYP isozymes might be due to the presence of aromatic nitrogen in the chemical structure of sildenafil, vardenafil, and tadalafil. It has been found that aromatic nitrogen group is the more likely inhibitor of cytochrome P450 isozymes due to its lipophilicity and ability to maintain hydrogen bonding with the CYP isoforms and decreasing their iron binding capacity [58].

NADPH-dependent cytochrome P450 reductase is a flavoprotein that catalyzes the reduction of CYPs using NADPH as a cofactor. The mitochondrial protein cytochrome c has been used occasionally as a model for analyzing the CYP reductase activity [56]. In addition, cytochrome b5 serves as an electron transfer intermediate between reductase and oxidative enzymes [59]. The present data showed that low and high doses of tadalafil, vardenafil, and sildenafil caused significant inhibition in the activity of NADPH-cytochrome c reductase which may consequently alter various metabolic and physiological pathways. The selective suppression of expression and activity of cytochrome P450 reductase under the influence of different doses of EDDs could lead to a number of metabolic changes in the liver tissue and may also affect the cytochrome P450-mediated drug metabolism [59]. In addition, mutations in the gene of cytochrome P450 reductase caused severe developmental malformations and disorder of steroidogenesis. These impairments were due to inhibition of the catalytic efficiency of 21-hydroxylation of progesterone, 17*α*-hydroxylation of progesterone, 17,20-lyase on 17-OH-pregnenolone, and aromatization of androstenedione [59]. Therefore, inhibition of the cytochrome c reductase due to the administration of tadalafil, vardenafil, and sildenafil to the male rats might affect these physiological pathways.

In conclusion, the present study showed tadalafil, vardenafil, and sildenafil changed the expression of various CYP isozymes (CYP1A2, 2B1/2, 3A4, 2C23, 2C6, and 2E1) and their associated metabolic activities. In addition, these changes may alter the metabolism of a wide range of xenobiotics as well as endogenous substrates that may lead to adverse effects of different xenobiotics.

## Figures and Tables

**Table 1:** Drug administration schedule of different EDDs.

Dose	Tadalafil (mg/Kg)	Vardenafil (mg/Kg)	Sildenafil (mg/Kg)
Low dose	0.280	0.28	1.43
High dose	1.43	1.43	7.15

All drugs were suspended in distilled water and the control group received only distilled water.

Low administered dose of these drugs was chosen according to FDA approval to human.

**Table 2:** Changes in the activities of phase I drug-metabolizing enzymes after treatment of male rats with low and high daily doses of tadalafil, vardenafil, and/or sildenafil for 21 days.

Parameters	Low dose treatments				High dose treatments			
	Control	Tadalafil	Vardenafil	Sildenafil	Control	Tadalafil	Vardenafil	Sildenafil
Cytochrome P450	0.94 ± 0.22	1.16 ± 0.36 (NS)	1.57 ± 0.48 (+67%)^*∗*^	1.43 ± 0.47 (+53%)^*∗*^	1.11 ± 0.36	0.22 ± 0.012 (−81%)^*∗*^	0.23 ± 0.012 (−79%)^*∗∗*^	0.234 ± 0.02 (−79%)^*∗*^
Cytochrome b_5_	0.69 ± 0.18	1.02 ± 0.24 (+47%)^*∗*^	1.11 ± 0.25 (+61%)^*∗∗*^	0.63 ± 0.16 (NS)	0.70 ± 0.27	0.33 ± 0.122(−53%)^*∗∗*^	0.212 ± 0.064 (−69%)^*∗∗*^	0.326 ± 0.015 (−53%)^*∗∗*^
Cytochrome c reductase	44 ± 7.03	35.4 ± 3.94 (−20%,)^*∗*^	29.8 ± 7.25 (−32%,)^*∗∗*^	30.55 ± 4.76 (−31%)^*∗∗*^	37.6 ± 9.2	17.9 ± 5.9 (−52%,)^*∗∗*^	12.6 ± 3.79 (−70%,)^*∗∗*^	15 ± 4.6 (−60%)^*∗∗*^
DMN-N-demethylase I	74.2 ± 8.28	98.1 ± 10.1 (+32%,)^*∗∗*^	91.7 ± 6.81 (+24%,)^*∗∗*^	80.4 ± 6.4 (NS)	65.7 ± 14.9	32.6 ± 9.2 (−50%,)^*∗∗*^	29 ± 6.1 (−56%)^*∗∗*^	31.3 ± 9.6 (−52%)^*∗∗*^
ECOD	1.31 ± 0.289	0.953 ± 0.219 (−27%,)^*∗*^	0.653 ± 0.22 (−50%)^*∗*^	1.245 ± 0.33 (NS)	1.16 ± 0.382	0.441 ± 0.144 (−62%)^*∗∗*^	0.779 ± 0.211 (−33%)^*∗*^	0.657 ± 0.12 (−43%)^*∗∗*^
EROD	14.36 ± 1.359	17.9 ± 3.61 (+25%)^*∗*^	15.62 ± 2.22 (NS)	10.28 ± 1.81 (−29%)^*∗*^	13.81 ± 2.8	8.42 ± 2.06 (−39%)^*∗∗*^	6.97 ± 1.48 (−50%)^*∗∗*^	9.25 ± 1.32 (−33%)^*∗∗*^
AHH	122.4 ± 25.82	151.3 ± 22.95 (NS)	320.9 ± 56.8 (+162%)^*∗∗*^	424.5 ± 72.89 (+247%,)^*∗∗*^	120.5 ± 34.2	77.5 ± 30.4 (−36%)^*∗∗*^	61.7 ± 15.3 (−49%)^*∗∗*^	52.3 ± 17.4 (−57%)^*∗∗*^

Cytochrome P450 (nmole CYP450/mg protein); cytochrome b_5_ (nmol cyt.b5/mg protein); cytochrome c reductase (nmole cyt. c reduced/mg protein/min); DMN-N-demethylase I (nmole HCHO/mg protein/h); ECOD (nmol 7-hydroxycoumarin/mg protein/min); EROD (nmole resorufin/mg protein/min); AHH (p mole 3-OH-B(a)P/mg protein/min).

Values are expressed as mean ± SEM of 10 rats in each group.

(*∗*) (*∗∗*) Values are statistically significant at *P* < 0.05 and *P* < 0.001, respectively; (NS): values are statistically nonsignificant.

## Abbreviations

ED: Erectile dysfunction
DMN-dI: Dimethylnitrosamine N-demethylase I
AHH: Arylhydrocarbon hydroxylase
ECOD: 7-Ethoxycoumarin-O-deethylase
EROD: Ethoxyresorufin-O-deethylase
PDE5Is: Phosphodiesterase-5 inhibitors
ROS: Reactive oxygen species
CYP: Cytochrome P450.
